# An Exploration of the Patient Lived Experience of Remission and Relapse of Type 2 Diabetes Following Bariatric Surgery

**DOI:** 10.1007/s11695-021-05514-7

**Published:** 2021-06-12

**Authors:** Alexis C. Sudlow, Dimitri J. Pournaras, Helen Heneghan, Zsolt Bodnar, Carel W. le Roux, Deidre McGillicuddy

**Affiliations:** 1 Department of Upper GI Surgery, Southmead Hospital, Southmead Road, Bristol, BS10 5NB UK; 2 Department of Surgery, St Vincent’s University Hospital, University College Dublin, Elm Park, Dublin, Ireland; 3 Department of Surgery, Letterkenny University Hospital, Donegal, Ireland; 4 Department of Experimental Pathology, University College Dublin, Belfield, Dublin, Ireland; 5 School of Education, University College Dublin, Belfield, Dublin, Ireland

**Keywords:** Obesity, Bariatric surgery, Type 2 diabetes mellitus, Patient-centred care

## Abstract

**Background:**

Bariatric surgery is the most effective treatment for patients with obesity and type 2 diabetes (T2DM), inducing profound metabolic changes associated with improvements in glycaemic control. In spite of the recognition of the physiological changes associated with bariatric surgery, what remains underappreciated is the patient experience of surgery to treat T2DM.

**Objectives:**

This study explored the patient experience with regard to motivations, expectations and outcomes, including remission and relapse of diabetes.

**Methods:**

An in-depth qualitative approach was adopted, encompassing semi-structured interviews with patients (n=17) living with obesity and T2DM both pre- and postsurgery. Interpretive thematic analysis identified emergent themes using a grounded approach.

**Results:**

Analysis revealed a number of themes throughout the interviews which included motivations and perceived benefits of surgery, obesity stigma and its impact on self-worth as well as perceptions of remission or relapse and the implications for sense of control.

**Conclusions:**

The motivation for undergoing bariatric surgery was driven by health concerns, namely T2DM and the desire to reduce the risk of developing diabetes-related complications. Patients highlighted social and self-stigmatisation associated with obesity and T2DM, leading to feelings of shame and an inability to seek support from family or healthcare professionals. Stigmatisation created a sense of failure and feeling of guilt for having T2DM. As a result, patients felt responsible for maintaining disease remission postoperatively and regarded the need for medication as a sign of treatment failure.

## Introduction

The treatment of type 2 diabetes (T2DM) remains one of the most common indications for bariatric surgery given the rapid normalisation of glucose homeostasis it induces [1, 2]. Although initially seen as a cure for T2DM, longer-term evidence has tempered this enthusiasm, replacing this view with a more realistic appreciation of bariatric surgery as a means of disease control. Our understanding of obesity and T2DM as chronic and progressive conditions suggests that the efficacy of treatment reduces over time and a proportion of patients may experience a relapse of diabetes.

Looking at outcomes in bariatric surgery, the focus is on weight loss, BMI, duration of disease remission and improvements in cardiovascular risk factors. While these are undoubtedly important metrics to consider, the experience of patients undergoing bariatric surgery to treat obesity and T2DM is often overlooked. It is widely understood that the desire to treat T2DM is one of the main reasons patients with obesity undergo bariatric surgery with most expecting disease remission. In spite of this, our insight into the patient experience, perceptions of the diagnosis of T2DM, remission and relapse is lacking, an understanding which is essential in delivering patient-focused care.

The aim of this study was to gain insights into patients’ lived experience with obesity, T2DM and bariatric surgery to inform the adoption of a patient-centred approach to treatment.

## Methods

This study employed an in-depth qualitative approach, encompassing semi-structured interviews with patients (n=17) living with obesity (BMI>30 kg/m^2^) and T2DM. Each patient was interviewed once using a semi-structured interview structure with open-ended questions focusing on several different domains the authors felt were important to understanding the lived experiences of patients with T2DM and obesity including motivations for undergoing surgery and perceptions of disease remission and relapse. Although the duration of the individual interview varied, a standardised format was followed to ensure all patients were asked the same questions. All interviews were carried out in English which was the first language of the interviewer and all participants. Interviews were conducted by the same person to limit variability in how they were conducted. Themes explored during the interviews included motivations for undergoing surgery, personal and social implications of living with obesity and T2DM and perceptions of diabetes remission and relapse. Phenomenological analysis was employed which is an approach to interpreting qualitative data whereby the researcher aims to capture the experience of several individuals through a reflective examination of their account or story [3, 4]. Thematic analysis involving critical review and coding of transcripts to identify common themes amongst responses was also carried out [5].

### Participant Recruitment

Selection criteria required a diagnosis of T2DM according to the ADA criteria, BMI >30 kg/m^2^ and that participants were awaiting or had already undergone bariatric surgery. Ethical approval was granted (HS-20-12-McGillicuddy). Purposive sampling was employed, identifying patients who would provide information-rich cases as well those able to communicate in a reflective manner [6]. Potential participants were informed that care would be in no way affected and they could withdraw at any point. The interviewer was not involved in the care of the participants at any point.

### Sample Size

There are no clear statistics-based rules for determination of sample size within qualitative research and the number tends to be deliberately limited in order to allow a focus on in-depth analysis in order to synthesise common themes and messages from a number of sources into a cohesive message [7]. This approach is supported by qualitative research methodologists who have suggested that the more information-rich data is collected from each person, the fewer participants are needed [8]. Moreover, sample sizes should be large enough to allow for an understanding of the phenomenon under investigation while also being small enough to allow for deep case-oriented analysis [9].

### Interview Structure

A semi-structured interview was employed, focusing on domains important to understanding the lived experiences of patients with T2DM and obesity. Interviews were conducted over the phone and digitally recorded.

### Analysis

A phenomenological approach to analysis was used with common themes identified to capture an overall understanding of the experience. Using an interpretive thematic approach required coding transcripts, identifying important issues which were indexed as they related to a theme. Specific statements were analysed and categorised into themes which related to the phenomenon being investigated [3]. Once themes were identified, transcripts were re-analysed to identify quotes which could be used for illustrative purposes [10].

## Results

### Participant Characteristics

Thirty-two patients met the eligibility requirements of which, and 17 agreed to participate (M=9, F=8). Fifteen (88%) had already undergone bariatric surgery of which 12 (80%) underwent RYGB and the remaining 3 had SG. Further patient characteristics can be found in Table 1.

**Table 1:** Patient characteristics

Age (median)	49 (SD=9.42)
Median preoperative BMI (kg/m^2^)	44.4 (SD=4.82)
Procedure performed (n=15)	RYGB= 80% (12) SG= 20% (3)
Preoperative treatment	Oral medication= 53% Oral medication + insulin= 47%
Postoperative treatment	Oral medication= 33.3% Oral medication + insulin= 0 No medication=66.6%

*BMI*, body mass index; *SD*, standard deviation

### Analysis

Analysis of the transcripts identified three important themes:

1. Motivation and perceived benefits of undergoing bariatric surgery
2. Implications of disease on self-worth, stigmatisation and psychosocial response
3. Perceptions of disease remission, relapse and control

Further analysis identified subthemes related to the major themes seen in Table 2.

**Table 2:** Main identified themes and related subthemes

Motivation and perceived benefit	• Fear • Functional limitation • Impact on family • Disease progression • Prevention of complications
Self-worth, stigmatisation and psychosocial response	• Shame • Judgement • Blame • Frustration • Failure • Personal responsibility for illness
Perceptions of disease remission, relapse and control	• Surgery to control disease process • Personal control over maintenance of remission • Self-efficacy • Cessation of medications

## Motivation and Perceived Benefits of Undergoing Bariatric Surgery

The main motivating factor for patients undergoing bariatric surgery was the diagnosis of T2DM. Although nearly all of the patients felt having T2DM negatively affected their quality of life, most felt this was secondary to concerns regarding the development of diabetes-related complications. Many felt without surgery, it was only a matter of time before complications manifested themselves.

I was very worried about my eyesight. I could lose my job if I had eyesight problems. I also knew people that lost toes and that really frightened me. (Richard, 40)

In addition to concerns about future health issues, many felt there was an imminent threat to their lives, adding a sense of urgency about undergoing bariatric.

I was getting really nervous about being so big because at that point, I was 150 kg. I was thinking if I don’t do something now to get this weight off, I could go with a heart attack tomorrow. (Michael, 42)

Most felt taking insulin would mark a deterioration in diabetes control. In those already taking insulin or patients where it had been considered, it intensified concerns regarding the impact of T2DM. Additionally, it prompted a reconsideration of bariatric surgery which some regarded as risky or extreme.

The diabetes was getting worse and they were saying about having take insulin. I just refused. I wasn’t going down that road with insulin. Because of that I thought I’d take my chance on something more extreme like an operation. (Kevin, 68)

Patients also considered surgery due to limitations in performing activities of daily living, low energy levels and physical pain which they hoped would improve.

I’ve got a son who is only 15 and a daughter who is 13. I realised I’ve never even played football with them. They’d only seen me huffing and puffing around the house because I was constantly so tired so that was a wake-up call. (Harry, 42)

Patients consistently expressed a fear of developing complications of diabetes as well as limitations regarding their ability to engage in activities of daily living as their primary motivation for undergoing surgery. The possibility of requiring insulin prompted patients to re-think the seriousness of their illness, a finding in keeping with studies demonstrating insulin use being seen as indicative of treatment failure and disease progression [11].

## Implications of Obesity and T2DM on Sense of Self-worth, Stigmatisation and Psychosocial Response

Patients frequently gave examples of overt and implied social stigmatisation relating to obesity, affecting their sense of self-worth. Participants felt others made assumptions or judgements based on weight alone and the implication being that obesity was the result of personal failings.

My biggest concern is what people think when they look at you. They think you’re somebody who is lazy or unable to control themselves or to self-indulgent, which may or may not be true. But it’s all decided by my physical appearance. (Grant, 46)

Experiences in the workplace were cited as examples of how obesity was often seen to define them as a person, making them a target for personal attacks.

I know that there are certain people that if I was told by somebody something at work and I questioned it, they would walk away and call me a fat cow. Because that’s how they see me. They think I have nothing other than that to offer. And people don’t consider being overweight, anything other than something you’ve chosen for yourself. (Marion, 48)

Patients also felt that their diagnosis of T2DM was seen by others as the result of personal failings.

If you’ve got type two diabetes, if you’ve had a heart attack or you’re overweight, people say....well you know you do to yourself, so no help should be there for you. You’re overweight so you sort it. (Ann, 49)

In addition to judgement as a result of having obesity and T2DM, patients appeared to internalise these messages and ultimately came to agree with them, resulting in shame with regard to their diagnosis and decision to undergo surgery.

I still am not happy about surgery and I’m not happy it’s a solution. I think it’s nuclear really in the scheme of things. And to be perfectly honest, I think it’s shameful as well. (Mike, 42)

Messages of blame and failure were deeply ingrained and firmly held. Even when presented with information to the contrary, many still felt having T2DM was due to a personal failing.

I absolutely feel responsible. And I’ve had this conversation with the doctor a number of times when he presents research and tells me the scientific perspective but I also know that if I eat better, exercise more and manage myself better, I would not be like this. I ultimately I make poor decisions. (Mike, 42)

Because of stigmatisation and self-blame, many patients attempted to hide their diagnosis of T2DM.

I don’t tell anyone, not even my parents know. People would certainly imply it’s my fault so it’s only my pharmacist and doctor who knows but outside the medical profession, nobody knows. (Bruce, 61)

Responses illustrated that obesity stigma remains one of the remaining socially acceptable forms of discrimination and is widespread. Although most participants recognise the multifactorial causes for the development of obesity, many have a firmly held belief that obesity is the result of personal failings.

## Patient Perception of Disease Remission, Relapse and Control

Patients held a paradoxical perception of their sense of control with regard to obesity and diabetes. Most believed bariatric surgery had become the only treatment option available having felt unable to maintain long-term weight control despite adherence to medical advice.

The weight was going up and no matter what, I just can’t seem to get control of it. Every time I tried to lose weight it was just it was coming back bigger and bigger than before. I felt I couldn’t control my weight or my diabetes. (Catherine, 45)

Moreover, they felt this differentiated them from peers without obesity who appeared to have control over their weight, producing a sense of frustration and hopelessness.

I have battled every day in my head and my all my life about my weight. I have thought, I am doing everything I can and why am I here? Why do I have to do so much more than my sister and my friends to lose weight? (Sarah, 46)

Regarding T2DM, patients expressed an inability to maintain glycaemic control, again despite adherence to dietary advice and pharmacotherapy. Even patients who had lost weight, some reported glycaemic control continuing to worsen.

For the first few years, you carried on taking more metformin. And then when I started losing weight because I’d been more mindful I suppose by then, the damage had been done. Every check-up was a wee bit stronger and there was talk of insulin. As the years went on and even though the weight went down, the blood sugars just kept going up and up. (Rachel, 48)

Although most expressed the sense they lacked control with regard to weight loss maintenance or glycaemic control, many felt the diagnosis of obesity and T2DM were their responsibility. This perception extended to the postoperative period whereby patients felt remission of T2DM was their responsibility and relapse would only occur due to a lack of adherence. Rather than viewing weight loss maintenance and diabetes control as an impossibility as it had been preoperatively, patients felt empowered and were confident in their ability to maintain control of weight and T2DM in the long term.

I don’t let myself think of relapse because of my lifestyle changes that are in place, that will never be an issue. (Andy, 51)

Patient perceptions of disease control or cure often equated to the cessation of all medications. Either all medications were stopped, and surgery was viewed as successful or they were continued/restarted in which case, the procedure had failed, even if glycaemic control had improved. In other words, success was measured in the discontinuation of medications rather than an improvement in glycaemia.

I would say restarting medications would be disastrous because it’s the primary purpose for doing this. (David, 46)

Most believed relapse would be solely due to personal failings rather than physiological processes. As an extension, many discussed the shame and disappointment they would feel if they experienced a relapse of T2DM or needed medication.

I’d be highly disappointed restarting medications but more feeling like I disappointed myself. Not the surgery was disappointing. (Ed, 51)

In addition to feeling personally responsible for maintaining remission of T2DM, many felt others would see it as being within their control to prevent relapse.

If my diabetes came back, it would be like an alcoholic drinking after a liver transplant. People would certainly judge you for not making the most of the surgery. (Maxwell, 49).

The perception of responsibility was clearly complicated with patients feeling a sense of a being unable to control their obesity or T2DM preoperatively followed by a sense of complete responsibility for maintenance of disease remission in the postoperative period.

## Discussion

Three interconnected but distinct themes emerged which were motivations and perceived benefits of bariatric surgery, implications of obesity and T2DM on sense of self-worth, stigma and psychosocial response and perception of disease remission, relapse and control.

Patients consistently reported their primary goal in undergoing bariatric surgery was to reduce their risk of developing diabetes-related complications. This finding is in keeping with previous studies demonstrating the decision to undergo bariatric surgery was driven by health concerns, independent of the degree of obesity and patients with T2DM were more likely to request surgery [12, 13]. Considering insulin use, patients not only viewed it as a sign of disease progression but one which marked a turning point, prompting a reconsideration of bariatric surgery.

Within many cultures, obesity is unfairly viewed as being the result of gluttony, lack of restraint and ultimately, personal failure. Conversely, prevention or disease treatment is achieved through moral fortitude and will power, requiring patients to eat less and exercise more. The biological plausibility of this approach combined with the observation most patients can achieve short-term weight loss neglects the reason why it is not an effective treatment for obesity. The challenge facing patients with obesity is not weight loss, rather weight loss maintenance. As a result, many feel those with obesity bear a personal responsibility and stigma is widespread, affecting patients in education, employment, healthcare and interpersonal relationships [14]. Experiences of stigma were common amongst participants and many internalised these messages resulting in self-stigmatisation, a process whereby individuals become aware of a stereotype, come to agree with it and ultimately, apply it to themselves resulting in reduced self-worth and self-efficacy. The implications of self-stigma were apparent with a widespread sense that weight and T2DM were beyond their control. Particularly with regard to T2DM, patients found even with weight loss and medical therapy, glycaemic control remained poor or even worsened. Despite feeling this was beyond their control, most continued to view this as a personal failure. Conversely, in the postoperative period, patients felt surgery had restored their sense of self-determination having previously felt helpless, burdened by a disease over which they felt they had no control. Although patients were aware of the possibility of disease relapse, none acknowledged it could be the result of disease progression or physiological factors rather than personal failing.

With any operation, it is essential both patient and surgeon agree regarding the aim of treatment. Studies suggest there is often a discord within bariatric surgery with differences in the priorities of patients compared to the surgeon negatively impacting patient perception of outcomes [15]. Participants consistently reported fears regarding imminent and long-term implications of T2DM and as such, this was consistently cited as a primary motivation for undergoing surgery. Although this is in concordance with the surgeon’s aim of treating T2DM to reduce the risk of developing diabetes-related complications, a dichotomy arises when one considers *how* each group quantifies or measures the success of surgery. From the patient perspective, poor diabetes control is evidenced by the need to take medications; thus, the continuation or the reintroduction of medication equates to the apparent failure of bariatric surgery as a means to control T2DM. This perception was evident in the responses of participants who suggested that the success of their surgery was reflected by the reduction or stoppage of medications. In spite of this, patients who remained on medications did acknowledge that although they were still taking medications, surgery had improved their glycaemic control and felt they had achieved their overall aim of reducing their risk of developing diabetes-related complication. The importance patients place on medication cessation as a surrogate marker of success stands in contrast to the point of view of the surgeon or physician, whereby a suboptimal surgical outcome is evidenced by ongoing poor diabetes control, quantified by measures of glycaemia. Given the mounting evidence supporting multimodal care, combining bariatric surgery with medications for long-term disease control, it is important to be mindful of this commonly held patient perception [16]. The way in which surgeons frame the goals of surgery has implications for patient willingness to accept further treatment such as pharmacotherapy to improve postoperative glycaemic control.

Although this study has limitations which should be recognised including the inability to conduct face to face interviews due to the COVID-19 pandemic and the potential lack of ethnic diversity in the population sampled given participants were drawn from only one country, it provides critical insight into the patient lived experience of undergoing bariatric surgery for T2DM, a previously unexplored topic. Additionally, given the use of a purposive sampling strategy to identify potential participants, there is the possibility of introducing a selection bias; however, patients were invited to participate based on their perceived willingness and ability to engage with the interview process, irrespective of their experience with the process of undergoing bariatric surgery, both positive and negative.

## Conclusions

An understanding of the motivations and experiences of patients undergoing bariatric surgery for the treatment of T2DM will help clinicians and patients align their goals and expectations, ultimately improving outcomes and the delivery of patient-focused care.
